# A psychology based approach for longitudinal development in cognitive robotics

**DOI:** 10.3389/fnbot.2014.00001

**Published:** 2014-01-27

**Authors:** J. Law, P. Shaw, K. Earland, M. Sheldon, M. Lee

**Affiliations:** Department of Computer Science, Aberystwyth UniversityAberystwyth, UK

**Keywords:** development, robotics, intrinsic motivation, staged learning, constraints

## Abstract

A major challenge in robotics is the ability to learn, from novel experiences, new behavior that is useful for achieving new goals and skills. Autonomous systems must be able to learn solely through the environment, thus ruling out *a priori* task knowledge, tuning, extensive training, or other forms of pre-programming. Learning must also be cumulative and incremental, as complex skills are built on top of primitive skills. Additionally, it must be driven by intrinsic motivation because formative experience is gained through autonomous activity, even in the absence of extrinsic goals or tasks. This paper presents an approach to these issues through robotic implementations inspired by the learning behavior of human infants. We describe an approach to developmental learning and present results from a demonstration of longitudinal development on an iCub humanoid robot. The results cover the rapid emergence of staged behavior, the role of constraints in development, the effect of bootstrapping between stages, and the use of a schema memory of experiential fragments in learning new skills. The context is a longitudinal experiment in which the robot advanced from uncontrolled motor babbling to skilled hand/eye integrated reaching and basic manipulation of objects. This approach offers promise for further fast and effective sensory-motor learning techniques for robotic learning.

## 1. Introduction

The question of autonomy poses a particularly hard challenge for robotics research—how can robots grow through the “open-ended acquisition of novel behavior?” That is, given an embodied robot system with some primitive actions, how can it learn appropriate new behaviors to deal with new and novel experiences. It is apparent that this will involve the integration of past experience with new sensorimotor possibilities, but this remains a difficult, important, and unsolved research area. We report on experiments that illustrate the value of a developmental attack on this issue.

Developmental robotics is a recent field of study that recognizes the role of epigenetic development as a new paradigm for adaptation and learning in robotics. Most research in this field reports on specific topics in development such as motivation, embodiment, enactive growth, imitation, self-awareness, agent interaction and other issues. Such investigations are exploring effective modeling methods and increasing our understanding of the many and varied aspects of the phenomenon of development. For general principles and reviews see Lungarella et al. (2003); Asada et al. (2009); Stoytchev (2009).

In our research, presented here, we place emphasis on two key features: the role of psychological theories in development; and the importance of longitudinal studies.

While all work in this field takes account of current knowledge in both neuroscience and experimental psychology, there exists a significant lacuna between psychological theories of development and our ability to implement those theories as working developmental algorithms. There is a large body of experimental work in psychology and we view psychological theory as a distillation of the understanding gained from such work that can guide modeling and help focus on key issues. In our work we are inspired by Piaget's extensive studies, particularly his emphasis on: staged growth; the fundamental role of sensory-motor development; and his constructivist approach (Piaget, 1973).

While recognizing that longitudinal development is a central issue, much current research has been focused on topics at particular stages in development, often involving cross-sectional data. This means that correspondingly less attention is being paid to the cumulative effects of continuous growth and the totality of the developmental trajectory. Some significant studies on longitudinal aspects have resulted in various time-lines or roadmaps being produced. These translate the developmental progression seen in human infants into a suggested or plausible trajectory of behavioral competence that might be expected of a successful robot model. Examples of roadmaps include that from the iTalk project (Cangelosi et al., 2010), the broad approach of the Jst Erato Asada project (http://jeap.jp/), and the output from the RobotCub project (Vernon et al., 2010). The work reported here is based on a detailed infant timeline (Law et al., 2011).

**Figure 1:**
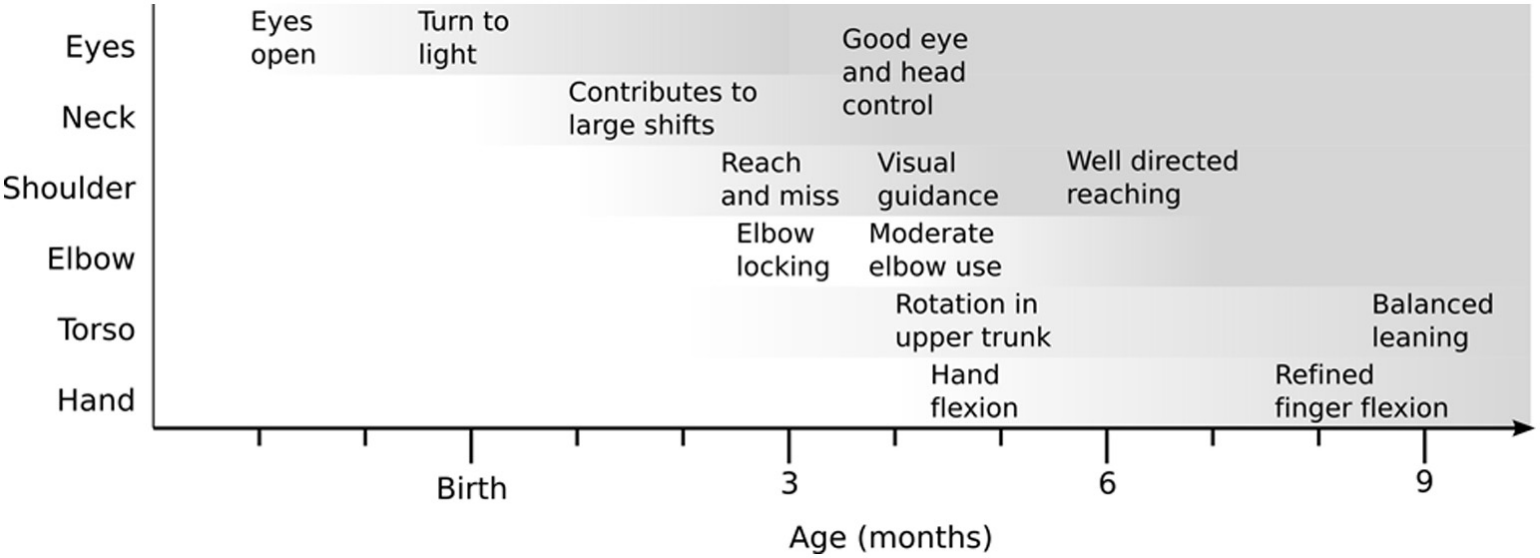
**A conceptual diagram of the increase in motor control competence in infancy, highlighting behaviors identified in the infant literature**. Darker shading indicates greater competency. This figure is abstracted from the detailed timeline compiled in Law et al. (2011).

This paper presents a model of longitudinal development analogous to part of the sensory-motor development of a human infant from birth to 6 months. Figure 1 gives an overview of the development timeline we use which is fully described in Law et al. (2011). We use an iCub humanoid robot (Natale et al., 2013) as the platform for our experiment. The robot is given no prior abilities and the task is to learn to coordinate and gain control of the motor system through some form of exploratory process. The criteria for success is in achieving sufficient competence to visually detect objects, reach toward and grasp them, and move them around in the environment. In other words, the aim is to advance from no understanding of the structure of the sensory-motor hardware to achieving skilled hand-eye coordination, involving reaching skills and mastery of the local egocentric space. To enable this, we provide the robot with a suitable architecture, on which to learn sensorimotor coordination, and a series of constraints designed to shape learning along a trajectory similar to that seen in infancy.

In this paper we present results from a complete longitudinal experiment that shows a full developmental cycle, progressing through several distinct behavioral stages and increasing competence from essentially no control (random motor action) to skilled visio-integrated reaching and manipulation of objects. This experiment was made possible by guidance from the results of several investigations into the various subsystems involved: eyes, head, arms, etc. While there is insufficient space to expand on all these prior studies, we reference them where appropriate in order to provide further background on particular aspects of our architecture. Particular new contributions include the use and control of the torso, reaching for objects, and schema learning for novel actions. The key findings reported here include: evidence for the speed and effectiveness of staged behavior; evidence for the role of constraints in staged development; the effect of bootstrapping between stages; and the use of a schema memory of experiential fragments in learning new skills. These are seen in the context of a longitudinal sequence showing the development in a continuous process—to our knowledge, this has not been performed on an iCub robot previously.

## 2. Materials and methods

**Figure 2:**
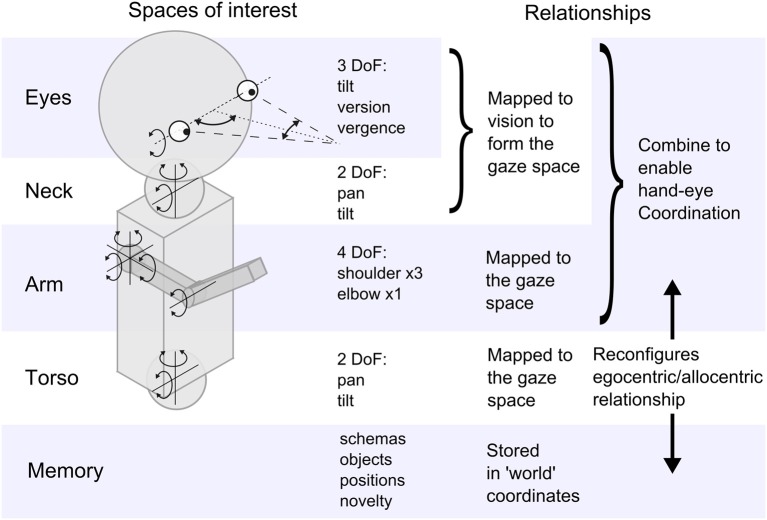
**A schematic representation of the iCub highlighting the sensorimotor spaces explored in the experiment, and the relationships between them**.

The experiments we describe here were performed on an iCub humanoid robot (Natale et al., 2013), depicted in terms of the sensor and motor systems of interest in Figure 2. The robot has a total of 53 independent degrees of freedom, however, here we only consider the 15 that are involved in hand/eye coordination (excluding the legs, hips, wrists and fingers). Although fine hand control (e.g., grasp adaptation to affordances) is not part of our study we do use some of the wrist and finger motors for simple hand closing reflexes.

The robot has joint angle sensors to provide proprioception and touch sensors in the hand that can simulate a primitive tactile sense. The eyes are color CCD cameras and so provide two 2D images, but the center of the retinal image is taken to be the loci of interest and the two eyes converge on a fixation point in a 3D visual space^1^. This visual space can be affected by several motor systems that cause bodily movement; for example if the head moves it will disturb the gaze point. However, the pattern of the disruption to vision is repeatable and lasting and so can be learned. This is shown in Figure 2 as a mapping process resulting in the *gaze space*—the space of visual fixation produced by the full range of eye and head movements. The gaze can move in this space without affecting the hands and vice versa and these two spaces must be related in some way to support hand/eye correlation and coordination. This is indicated as another mapping. Movement of the torso affects both hands and eyes and the resulting disturbance effects must be similarly mapped onto the gaze space. Figure 2 also indicates that memory will be necessary to record learning of significant and successful experiences, and we use a schema formalism for this.

Given this anatomy we can now define the initial state of the system prior to the experiment. The robot will be furnished with a framework upon which to learn hand-eye coordination and object interaction. This framework will support learning in the various sensor and motor modalities, coordination between modalities, and the creation and integration of schemas. Initially it will not contain any schemas or data on the coordination of sensor and motor systems, with this being learnt through exploration and interaction.

The goal of the experiment is for the robot to progress from the initial state to a state where it has control over its subsystems so that hand-eye coordinated reaching is achieved. We can measure attainment of the goal by an ability to reach for objects and to move them in the environment. A second objective is to achieve this through a process of novelty-driven learning that models the development shown by infants. This means learning must not involve supervision, yet it must also be constrained within an acceptable rate for real robot systems.

### 2.1. Design of the experimental architecture—intrinsic motivation

There are several key concepts implemented in our experimental software that form the basis of our approach. These include a motivational mechanism, a staged learning framework, a spatial sensorimotor substrate, and a schema memory for the recall and generalization of previous experiences.

The first concern is intrinsic motivation: as our robot is not given any goals or tasks, how (or even why) can it perform actions? Extrinsic goals are not sufficient to explain all behavior—some behavior is essentially internally driven, and this is particularly significant for the developing infant. For example, in a quiescent state with no external demands or priorities there may be a range of possible actions available but no indication or experience of the outcomes of those actions. In such cases many robotic projects have used the idea of “motor babbling” to select the next action randomly, e.g., Caligiore et al. (2008) and Saegusa et al. (2009). This relates to an enactive view of cognition in which action is seen as part of sensory data gathering. An interesting line of investigation is the work of Kuniyoshi and Sangawa (2006), who study and simulate the class of general movements in the fetus as a bootstrapping stage for postnatal exploitation.

Following Bruner et al. (1976), we use novelty as a driver. Novel events that can be repeated and possibly correlated with other events are given high saliency. We prefer a broad and general definition of novelty that can be widely scientifically applicable. So our mechanism for novelty is simply to define any new event as stimulating. This is very general in that it includes new external stimuli, new internal experiences (such as from muscles or proprioception), new forms of interaction, or new sequencing of known events. Whether an event is detected as new by the robot, depends on it being sufficient to be detected by the sensing abilities of the system, and whether or not it was predicted, i.e., had a prior representation of it being experienced before^2^. For example, a visual stimulus will be detected as new because it appears in a new location, or has changed color (provided neither were predicted). A movement of the robot will be considered new if it results in a detectable position that has not been previously encountered. An action combination will be considered new if it results in a change of world state that was not predicted. As an event or perceived structure becomes familiar so it will no longer be novel and becomes of less interest. This means the scope of novelty will change and evolve: initially even basic movements of the body parts are novel but later on objects become more interesting, followed by interactions with moving objects, animated objects and people.

In our implementation we assign all distinct stimuli, objects or other sensed entities, with an excitation variable that is given a high value on first encounter. All excitations decay with time and a habituation function provides a brief sensitization period followed by a decline in excitation on repeated stimuli. This excitation regime provides a saliency device and a winner-takes-all selector then acts as an attention mechanism. With this arrangement the focus of attention is attracted toward the items that have been the most novel most recently. Over time the decay function will cause past events to be forgotten, thus effecting a short-term memory, and it then becomes possible for an old experience to become stimulating again.

When attention is attracted to a novel stimulus then activity is initiated in the form of motor babbling. Sometimes the stimulus will have no further existence but sometimes an action may co-occur with a repeat of the stimulus. In our approach, a major assumption is based on repeated events; if a novel stimulus can be repeated when a given action is performed then the stimulus and the action are likely to be causally linked (Lee et al., 2007). Hence, repetition is an important part of motor babbling. When a babbling action apparently disturbs a sensory signal of interest then the system is strongly motivated to repeat the action, and if the effect is confirmed then an association can be recorded in the developing perceptual structures. This form of correlation is correspondence-based with tight temporal constraints for simultaneous events—in neuroscience the window for events that are perceived to be the same or connected is reported to be lower than 10 ms (Caporale and Dan, 2008; Markram et al., 2011). For actions that need to be completed before their effect can be correlated with a stimulus we note that the basal ganglia uses dopamine effects for identifying which of several ongoing actions is the correlating action (Redgrave et al., 2013).

**Figure 3:**
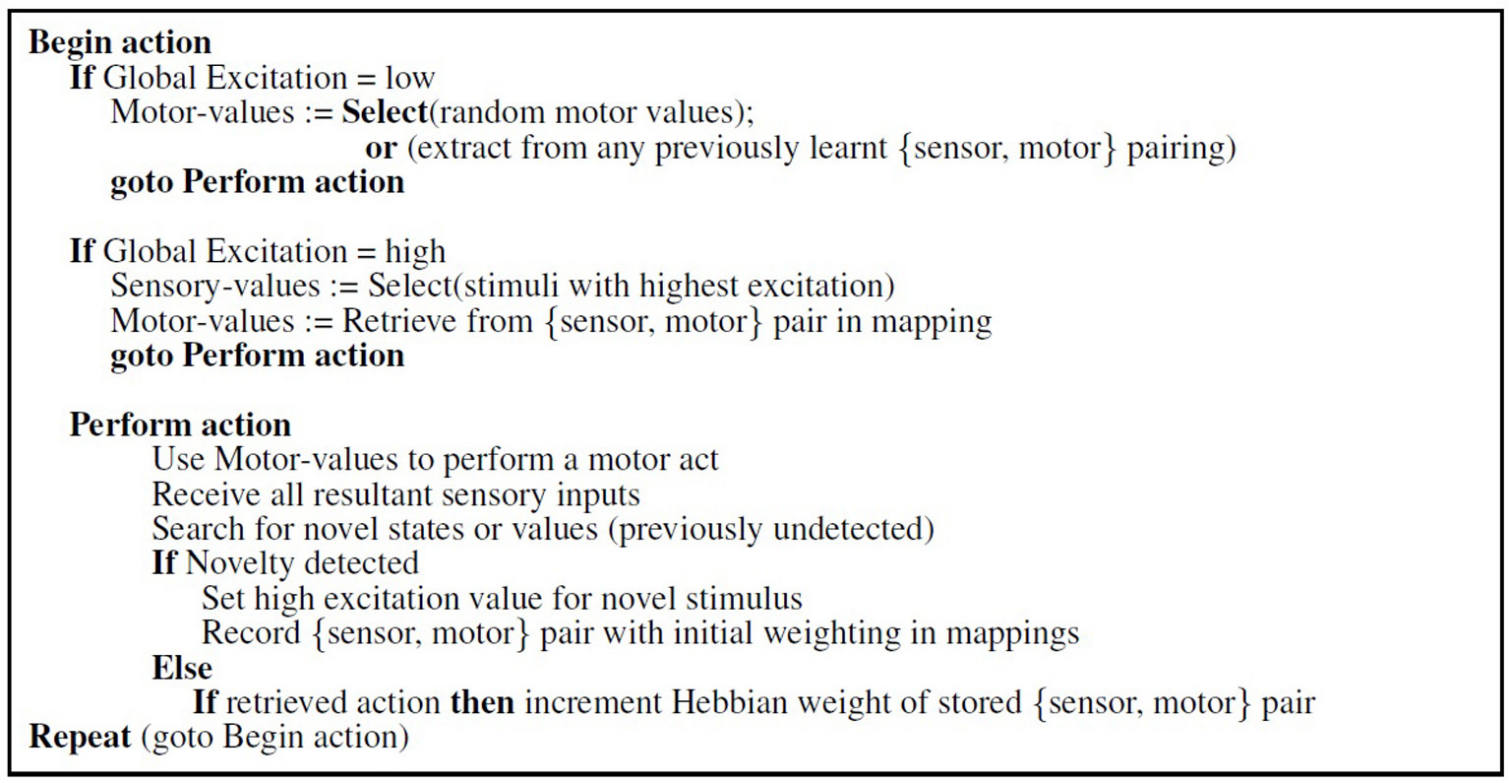
**Algorithm for novelty-driven action selection (derived from experiments in Law et al., 2011)**.

Figure 3 shows the algorithm for this process. Motor action is driven by novel stimuli, with correlations (mappings) between sensor and motor pairings being reinforced through repetition. Global excitation is the summation of all the excitation variables and is used to select motor babbling when there has been a period with no novel events.

In our systems novelty usually comes in the form of an unexpected sensory stimulus (visual, tactile, audio, etc.) or a stimulus that correlates with a motor act (arm movement and proprioception, hand movement and visual regard, object contact and movement, etc.). In the former case the saliency mechanism uses excitation values to select the most novel stimuli to attend to. There is no threshold limit: the highest excitation wins. In the latter case, the algorithm compares sensor and motor pairs with those stored in memory. If the new event is not already stored, then it is deemed novel and selected for further exploration. If it is repeatable and temporally coincident, it becomes saved as reliable experience.

### 2.2. Task learning complexity

The second design issue concerns the complexity of the task of learning how a many degree of freedom system is related and structured. This becomes very difficult and computationally expensive for high orders and for our 15 DoF robot it is impracticable to consider learning over all the motor systems at once. However, infants face an identical problem, and they solve it incrementally and in real-time. Infant development is characterized by the phenomenon of staged behavior, during which prominent sequences are readily observed, for example, sitting, crawling, and walking. Competence in a task is preceded by mastery of other subtasks, and such stages involve periods of learning followed by consolidation (Piaget and Cook, 1952). Transitions between stages are neither instantaneous nor absolute, as one pattern of behavior supersedes or merges into another as the underlying control schemas change (Guerin et al., 2013). Piaget's theories were extended by Kalnins and Bruner (1973) and Bruner (1990), suggesting mechanisms that could explain the relation of symbols to motor acts, especially concerning the manipulation of objects and the interpretation of observations.

Table 1 has been derived from the developmental literature and shows the sequence of development of motor control for the period up to 9 months. It shows the cephalocaudal direction of development, beginning with the eyes and head, and flowing down through the arms, hands, and torso. Early grasping and ungrasping, appearing before birth and at 2 months, respectively, are reflexive, but are included here as they provide vital actions for the development of behaviors. They enable the infant to perform basic manual interaction, and thus gain additional sensory information, without having to wait for controlled grasping to appear. These early, reflexive, actions are likely to help bootstrap later behavior, and highlight the importance of the concept of staged development: that it significantly reduces the complexity of the learning task.

**Table 1:** **Infant development and learning targets**.

**Age (months)**	**Observed behavior**	**Robot targets**
Pre-natal	Grasp reflex Butterworth and Harris, 1994	Grasp on tactile feedback
1	Sufficient muscle tone to support brief head movements Fiorentino, 1981	Constraint on head movement
1	Eyes and head move to targets Sheridan, 1973	Learning of saccade mappings
1	Saccades are few in number Maurer and Maurer, 1988	
2	More saccades Maurer and Maurer, 1988 and improved control Fiorentino, 1981	Refinement of saccade mappings
2	Head only contributes to larger gaze shifts due to lack of muscle tone Goodkin, 1980	Release of constraint on head motion, and beginnings of eye-head mapping
2	Involuntary grasp release Fiorentino, 1981	Release grasp when hand attention is low
3	Head contributes to small gaze shifts 25% of the time, and always to large gaze shifts Goodkin, 1980	Refinement of eye-head gaze control
3	Reach and miss Shirley, 1933 with some contacts Fiorentino, 1981	Reaching triggered by visual stimulation
3	Hand regard and hands to mouth Fiorentino, 1981	Initial learning of eye-hand mappings with return to “home” position
3	Clasps and unclasps hands Sheridan, 1973	Learning of raking grasp
4	Good eye and head control Fiorentino, 1981	Gaze mapping completed
4	Beginning thumb opposition Bayley, 1936	Enable independent thumb movement
5	Rotation in upper trunk Fiorentino, 1981	Begin torso mapping
5	Palmar grasp Fiorentino, 1981	Learning of palmar grasp
6	Successful reach and grasp Sheridan, 1973	Refinement of visually-guided reaching
7	Thumb opposition complete Bayley, 1936	Refined thumb use
8	Pincer grasp, bilateral, unilateral, transfer Fiorentino, 1981	Learning of pincer grasp
8	Crude voluntary release of objects Fiorentino, 1981	Voluntary release
9	Leans forward without losing balance Sheridan, 1973	Torso mapping complete

Table 2 shows a similar set of data specifically for the behavioral stages identified in the development of reaching. We note that early reaching is driven by tactile and proprioceptive feedback, before vision is well established. As vision improves, so too does the level of involvement of vision in the feedback process: early arm movements are triggered by visual stimuli; the first successful reaches are visually elicited, with the eyes fixated on the target and not the hand; later reaches use visual feedback to reduce the error between the hand position and the target. There is also an element of proximo-distal development, with control of the shoulder appearing before the elbow and hands.

**Table 2:** **Reach development and learning targets**.

**Age (months)**	**Observed behavior**	**Robot targets**
Pre-natal	Arm babbling in the womb De Vries et al., 1984	Proprioceptive-motor mapping of general movements
1	Hand-mouth movements Rochat, 1993	Learning of home position through tactile feedback
1	Directed (to the hemifield in which a target appears), but unsuccessful, hand movements von Hofsten and Rönnqvist, 1993; Ennouri and Bloch, 1996	Initial mapping of general movements to vision
1	Initial reaching is goal directed, and triggered by a visual stimulus, but visual feedback is not used to correct movements mid-reach Bremner, 1994, p. 38	Visual stimuli trigger general reach movements
3	Infants often move their hand to a pre-reaching position near the head before starting a reach Berthier et al., 1999, which then follows the line of sight Bruner, 1968, p. 44	Reaches conducted from “home” position
3	Infants engaged in early reaching maintained a constant hand-body distance by locking the elbow, and instead used torso movements to alter the distance to targets Berthier et al., 1999	Constraints on elbow movements reduce learning space
3	Successful reaching appears around 3–4 months after birth Shirley, 1933; Fiorentino, 1981; Berthier et al., 1999; Berthier and Keen, 2006	Primitive hand-eye mapping
3	Gaze still focused on the target and not the hand Clifton et al., 1993; Butterworth and Harris, 1994; Clifton et al., 1994; Berthier and Carrico, 2010	Reaches are visually elicited, but without continuous feedback
4	From 4 months, infants begin to use visual feedback to refine the movement of the hand White et al., 1964	Begin to map joint-visual changes and use visual feedback to correct reaches
4	As infants age their reaching becomes straighter, with the hand following the shortest path Carvalho et al., 2007	Refined reaching with smooth and direct movements

Together with the infant behaviors are a suggested series of stages that a robot could follow to achieve the same performance. These have been generated by relating the infant data to the specification of the iCub robot. However, they are general enough as to be applicable to most humanoid robot platforms. In the experiment described here we aim to reconstruct the first 5 months of development indicated by these tables as a series of behavioral stages on the iCub robot.

The phenomenon of staged growth has been linked to the existence of maturational or environmental constraints. Various forms of constraint can be identified that restrict the range of sensorimotor functionality available to the young infant. One example of underlying constraints is seen in the development of the newborn that proceeds in a cephalocaudal manner, with behaviors emerging sequentially down through the body and including looking, orienting, swiping, reaching, grasping, standing, and walking. We modeled these effects in our robot experiments by restricting the information and action possibilities available to the robot; thus the complexity of the learning space is reduced, with related restrictions on the behaviors produced. In particular, we focus on how maturational constraints and individual experience affect the emergence of stages. In our robotic systems constraints can be structured (Type A), or emergent (Type B). Type A constraints are analogous to maturation in neurological and physiological structures, and cover changes in myelination, sensory resolution, muscle tone, etc. In contrast, Type B constraints emerge from interactions and experience. As the infant/robot develops, both types of constraints can be released, through maturation or interaction, leading to new abilities and behaviors.

Type A constraints are considered to be hard constraints on the developmental trajectory due to the physical growth or maturity necessary for their removal. Individual infants develop at different rates, making timings of constraint releases difficult to define, however, the trajectories tend to be similar, following a regular sequence of stages. A timeline is presented in Law et al. (2011) and can be applied to a developing robot by using internal state variables as the indicator to trigger removal of constraints in a semi-structured manner (Lee et al., 2007). This will cause the robot to follow the general infant trajectory, where the timings of constraint release are based on its own individual circumstances.

Type B constraints are caused by external factors that effect development, such as the level of stimulation in the environment or the amount and form of interaction with carers. The strong influence of these factors on the order in which development occurs has been recorded in observation and demonstrated in various experiments. For example the use of a “sticky mitten” to compensate for the lack of competence in grasping, facilitated infants with a precocious and greater level of manual interaction with objects (Needham et al., 2002).

Both types of constraint play an important role in this experiment. The development of muscle tone, a Type A constraint, is cited as a driver for cephalocaudal development, and provides us with our basis for creating the pattern of behavior in Table 1. As we are not able to accurately model this type of development, we simulate it as a series of constraints preventing movement at each set of joints. These constraints are released in sequence, starting with the eyes at the outset, and progressing down the body as the experiment continues. Whereas muscle tone is likely to be related to age, our constraints are related to level of ability, as our developmental sequence has a much shorter time scale than that of the infant.

Other Type A constraints, in the form of sensory availability and resolution, are used to shape reaching actions. Initial arm movements are formed using tactile and proprioceptive feedback without any visual information. Once vision is active, it can be incorporated into reach learning, but resolution in the infant gradually increases, and we model this growth. Early visually triggered reaches generate very coarse visual stimuli, so result in inaccurate swiping behaviors in the general direction of objects. As vision and gaze control improve, so does the quality of reach. However, visual feedback is not enabled to guide reaching until visually elicited reaches have become successful. Due to the requirement for physical interaction during reach learning and the need to avoid potentially harmful robot actions, we conduct these stages in simulation and transfer them to the robot when accurate reaching has been achieved.

In addition to these Type A constraints, our experiment relies on Type B constraints arising from the environment. Although the effects are often quite subtle they can also be quite pronounced, for instance the number and positioning of stimuli impact on the extent of learning. Due to the size and nature of the experiment these influences make it very difficult to measure their effects or replicate data precisely. To show the impact of these constraints we investigate how changing the environment affects learning of gaze control. Details of the constraints used in this experiment are given in Tables 3, 4. In Table 3, the Type A constraint on the torso and arm learning is the same, i.e., restriction of movement due to immaturity. However, if these two components tried to learn in parallel then a number of variables and unconstrained degrees of freedom would be active at the same time. It would be very difficult to identify which motor movements caused which effects, making it very difficult to learn anything meaningful. As a result, the constraints are used to prevent them learning at exactly the same time, but the order in which they learn is flexible being based on stimulation and events in the environment. Consequently, we label these as containing both type A and B constraints. It is equally possible that the two could alternate in their learning, with constraints being intermittently applied to alternating components. Neck learning is also shown in Table 3 as Type B; this is because neck learning does not need a threshold or trigger as it is only effective when eye control is well developed. Hence, it can be permanently enabled but will only emerge as and when the eye system achieves sufficient competence. Such emergence is typical of Type B constraints.

**Table 3:** **Constraints used to structure behavioral stages on the robot**.

**Constraint**	**Type**	**Effect**	**Removal trigger**
Environment	B	Affects data available for learning at all stages	None. Influenced by robot and experimenter
Eye motor	A	Prevents eye motion	Start of experiment
Neck motor	A	Prevents head motion	Threshold on eye control
Neck learning	B	Neck learning requires accurate eye control	Emerges as eye control develops
Shoulder motors	A + B	Prevents arm movement	Threshold on gaze control, exclusive of torso learning
Elbow motors	A + B	Limits forearm extension/flexion	Threshold on gaze control, exclusive of torso learning
Reflex grasp	A	Causes hand to close on tactile stimuli	Active until reaching threshold attained
Controlled grasp	A	Prevents voluntary grasping of objects	Released with shoulder
Torso motor	A + B	Prevents motion at waist	Threshold on gaze control, exclusive of arm learning

**Table 4:** **Constraints used to structure reaching stages in simulation**.

**Constraint**	**Type**	**Effect**	**Removal trigger**
No vision	A	Arm movements learnt through tactile and proprioceptive feedback only	Start of experiment
Crude gaze fields (large)	A	Arm movements coarsely correlated with vision	Threshold on maturity of internal structures
Fine gaze fields (small)	A	Fine correlation between hand position and vision	Threshold on development of reaching
Visual feedback	A	Prevents visual guidance during reaching	Threshold on successful reaching

### 2.3. Mapping technique

**Figure 4:**
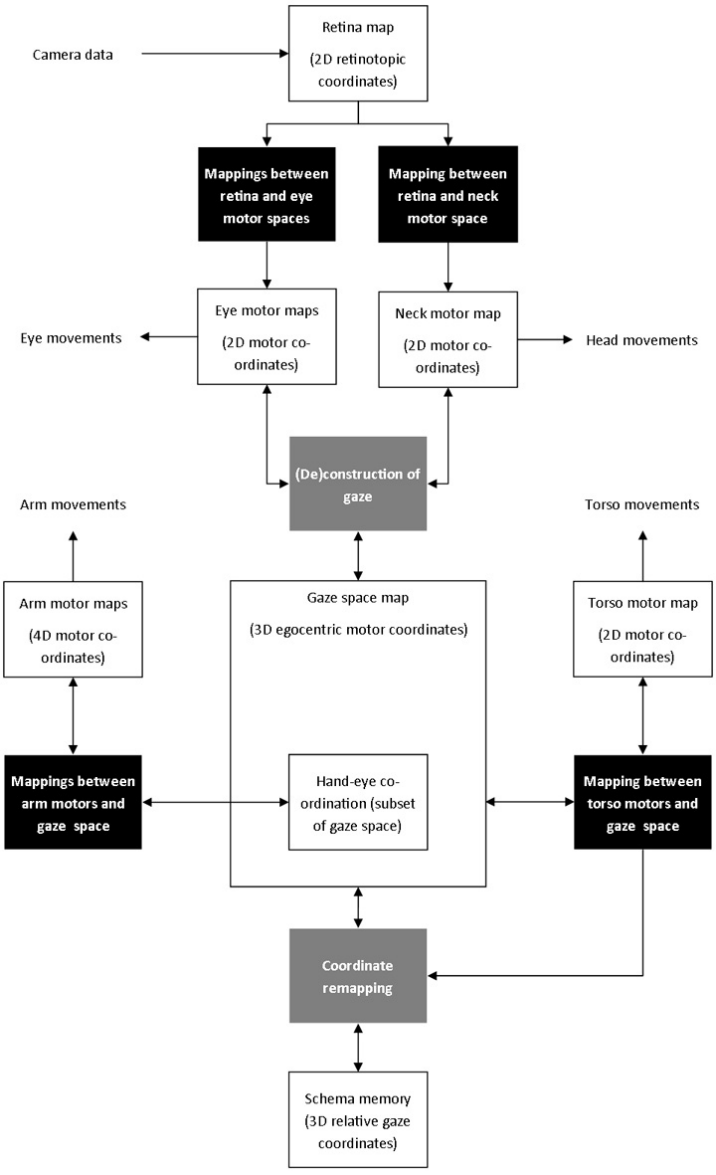
**System architecture**.

The third key issue concerns the design of a suitable computational substrate that will support the representation of whatever sensorimotor structure is discovered by experience. This involves spatial data as can be seen from the robot hardware in Figure 2. This figure suggests the fundamental spaces produced by the sensory-motor configuration of the iCub robot and, following the embodiment principle, this will vary for different anatomies. Considering the staged organization mentioned above, we designed the architecture shown in Figure 4 to capture the relations and mappings indicated in Figure 2.

Learning data in this experiment is based on visual and proprioceptive data. That is, the image data collected by the two cameras, and the information from the position of each joint. Tactile sensors trigger reflexive grasping, but are not directly used in learning. The main components of the architecture are as follows:

- Visual stimuli on the camera sensor are encoded on a 2D retinotopic map and linked to 2D motor maps for the eyes and neck. These enable the robot to learn the correspondence between moving the eyes and neck, and movements of visual stimuli. A mechanism for gaze control based on biological data interacts with both the eye and neck motor maps to generate stereotypical gaze shifts. The combination of both eyes and neck displacements defines the gaze space—the 3D egocentric model of space used to coordinate the robot's actions (see Law et al., 2013 for further details).
- 4D arm motor movements are mapped to a portion of the gaze space, for hand-eye coordination.
- 2D torso motor movements are mapped to the gaze space to define how body movements affect the movement of visual targets.
- The memory schema records the positions and details of objects in the 3D gaze space, but the relationship between the current gaze direction and the remembered positions changes as the robot moves. Data from the learnt torso mapping is used to transform remembered positions into relative gaze positions.

The architecture is thus a cross-modal representation of the robot and its personal space. At the core of our architecture is the 3D egocentric gaze space, which maps the proprioceptively-sensed gaze direction of the eye and head to the visual space of the retina and the proprioceptively-sensed position of the limbs in joint space. This building up of an internal body model from a collection of smaller spaces has been investigated by others, e.g., Morasso and Sanguineti (1995) and Fuke et al. (2009) but the key challenge is in keeping the computational demands of the techniques within the bounds of biological plausibility.

Piaget suggested infants first construct an egocentric representation of space through sensorimotor interaction, and that this gradually gave way, over the first year of life, to the ability to locate objects in relation to external landmarks (Piaget and Inhelder, 1956). More recently, this has given way to the idea of infants developing an allocentric representation of space, based on a variety of coding mechanisms (Newcombe and Huttenlocher, 2006). This shift is most noticeable in the latter half of the first year (Acredolo and Evans, 1980; Newcombe and Huttenlocher, 2006; Vasilyeva and Lourenco, 2012), beyond the period of our current investigation, but has also been suggested to appear as early as 4 months (Kaufman et al., 2006; Bremner et al., 2008). The shift from egocentric to allocentric representation is noted to be slow, and could be related to a number of factors including identification of visual landmarks, rotation of the torso, and crawling (Newcombe and Huttenlocher, 2006; Vasilyeva and Lourenco, 2012), and that it could be impaired by cognitive load (Kaufman et al., 2006). Our vision system is not capable of identifying relationships between objects, nor does the robot perform any relocation of the body until the torso develops late in the experiment. Therefore, we currently restrict our model to the early egocentric and proprioceptive representation of space.

Motor babbling generates candidate data for learning this sensorimotor coordination. The discovered associations between stimuli properties and motor acts represent important information that will support further competencies. For example, in controlling the eyeball to move to fixate on a target it is necessary to know the relationship between the target distance from the center of the retina and the strength of the motor signals required to move the eye to this point. As targets vary their location in the retinal periphery so the required motor command also varies.

We use a mapping method as a framework for sensorimotor coordination (Lee et al., 2007). A *mapping* consists of two 2D arrays (or *maps*), representing sensory or motor variables, connected together by a set of explicit links that join points or small regions, known as *fields*, in each array. Although three dimensions might seem appropriate for representing spatial events, we take inspiration from neuroscience, which shows that most areas of the brain are organized in topographical two-dimensional layers^3^ (Mallot et al., 1990; Braitenberg and SchüZ, 1991). This remarkable structural consistency suggests some potential advantage or efficacy in such two-dimensional arrangements (Kaas, 1997).

Fields are analogous to receptive fields in the brain, and identify regions of equivalence. Any stimulus falling within a field produces an output. A single stimulus may activate a number of fields if it occurs in an area of overlap between fields. Further studies of the map structure and how it relates to neural sheets in the brain is presented in Earland et al. (2014).

For the saccade example, a 2D map of the retina is connected to a 2D array of motor values corresponding to the two degrees of freedom provided by the two axes of movement of the eyeball (pan and tilt). The connections (representing the mapping) between the two arrays are established from sensory-motor pairs that are produced during learning. Eventually the maps are fully populated and linked, but even before then they can be used to drive saccades if entries have been created for the current target location.

Mappings provide us with a method of connecting multiple sensor and motor systems that are directly related. This is sufficient for simple control of independent motor systems, such as by generating eye-motor commands to fixate on a particular stimulus. However, more complex and interdependent combinations of sensor and motor systems require additional circuitry and mechanisms in order to provide the required functionality. For example, audio-visual localization requires the correlation of audible stimuli in head-centered coordinates, with visual stimuli in eye-centered coordinates. The system has the added complexity that the eye is free to rotate within the head, making a direct mapping between audio and visual stimuli impossible. Just as in the brain, careful organization and structuring of these mechanisms and mappings is required.

To control coupled sensorimotor systems, such as the eye and head during gaze shifts, we take inspiration from the relevant biological literature (Guitton and Volle, 1987; Goossens and van Opstal, 1997; Girard and Berthoz, 2005; Freedman, 2008; Gandhi and Katnani, 2011). Our aim is to reproduce the mechanisms at a functional level, and connect them to form an appropriate abstraction of their biological counterparts. We do not endeavor to create accurate neurophysiological models but rather to create plausible models at a functional level based on well-established hypotheses. Consequently we use mappings to transform between sensorimotor domains, and incorporate standard robotic sensors and actuators, and low-level motor control techniques in place of their biological equivalents.

### 2.4. Sensorimotor memory

The final design issue concerns the requirement to remember learned skills. The system as described above has a rich sensorimotor model of the immediate events being experienced but has limited memory of these experiences.

Until this point the mappings have acted as the sole memory component, storing both short term sensory, and long term coordination information. The sensory events are mainly spatially encoded, in the robot's “egosphere” as indicated above, and these have short term memory—when their excitation decays they may be experienced again as “new” events. On the other hand, the coordinations between motor and sensory subsystems are stored as connections and thus represent long term memories (with scope for plastic variation). These are also mainly spatially encoded experiences and so represent, for example, how to reach and touch an object seen at a specific location. What is not represented is any sensorimotor experience that has temporally dependent aspects. For example, consider a sequence of actions such as: reach to object, grasp object, move to another location, release object. This can be seen as a single compound action (move object) consisting of four temporally ordered actions.

For this reason we introduced a long term associative memory mechanism that supports: the memory of successful basic action patterns; the associative matching of current sensorimotor experience to the stored action patterns; the generalization of several similar experiences into single parameterized patterns; and the composition of action chains for the temporal execution of more powerful action sequences. A concomitant feature of such requirements is that the patterns in long term memory should be useful as predictors of action outcome—a function that is unavailable without action memory. Inspired by Piaget's notions of schemas (Piaget and Cook, 1952) we implemented a schema memory system that stores action representations as triples consisting of: the pre-conditions that existed before the action; the action performed; and the resulting post-conditions (see Sheldon, 2012 for further details). The schema memory provides long term memory in order to prevent repeated attention on past stimuli, and to match previous actions to new events. This formalism has been used by others, e.g., (Guerin et al., 2013), and is a flexible and general representation that allows extensions and supports all the above requirements.

## 3. Results

There are four significant results from this longitudinal experiment.

### 3.1. Emergence of developmental stages

The first result concerns the emergence of a series of distinct qualitative stages in the robot's behavior over the duration of the experiment. The results described here used maturity levels for constraint release that previous experiments suggested as reasonable. This gave competence for reaching to be performed with an end-point accuracy of 1 cm, which is sufficient for the iCub to grasp 6–8 cm objects. Further data on the effects of staged constraint release can be found in Shaw et al. (2014).

**Figure 5:**
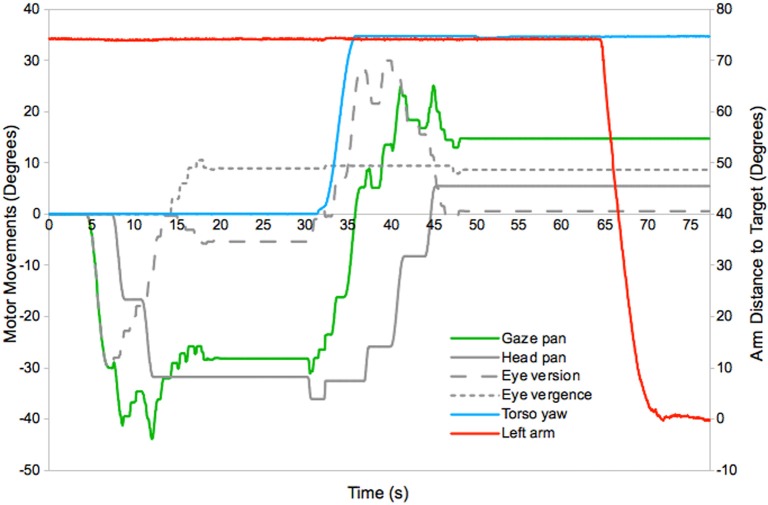
**Motor dynamics in the horizontal plane during a typical gaze and reach action (see text for details)^4^**.

An example of the motor dynamics exhibited during reaching, using maps learnt in this experiment, is given in Figure 5. This shows the use of the eyes, head, torso, and arm joints to gaze to a novel target, bring it into reach, and place the hand at its location. At around 4 s into the experiment a novel target appears and the robot initiates a gaze shift. This is produced using the eye and head motor movements mapped to the location of the stimulus in the retina map. The eye is the first system to move, and fixates on the target at around 6 s. The head then begins to contribute to the gaze shift, and the eye counter-rotates to keep the target fixated (the mapping resolution and dynamics of the system result in jerky head movements and some fluctuation of the gaze direction). The gaze shift completes at 14 s and is followed by a separate vergence movement to determine the distance to the target (this has a small effect on the gaze direction, which is based on readings from the dominant eye). Full fixation occurs around 19 s. Next the robot selects a torso movement to position the target within the reach space. This takes place between 31 and 35 s and is accompanied by compensatory eye and head movements, which complete at 48 s. Finally arm movements are triggered at 65 s, which result in the hand arriving at the target at 71 s.

Table 5 records these stages and also their rapid rate of development. As previously explained, the early reaching actions are first learnt in simulation before being integrated with other actions learnt on the real robot. Aside from this deviation for safety purposes, all actions are learnt on-line on the robot. All actions learnt in simulation are performed at a speed equivalent to real-time on the physical robot in order that the results are comparable^5^.

**Table 5:** **Observable experimental behaviors**.

**Behavior**	**Description**	**Duration (min)**	**Platform**
Fetal babbling	General arm movements	10	Simulator
Saccading	Eye movements only, trying to fixate on stimuli	20	Robot
Gazing	Eyes and head move to fixate on stimuli	40	Robot
Swiping	Arms make swiping actions in the general direction of visual stimuli	10	Simulator
Visually elicited reaching	Reaches toward visual targets with some success	10	Simulator
Guided reaching	Successful and smoother reaches toward visual targets	60	Both
Torso movement	Moves at waist to reach objects	20	Robot
Object play	Grasps objects and moves them around	40	Robot

Tables 6, 7 expand on the detail and show the time point when each stage was observed to first appear.^6^ The resultant behavior patterns are similar to those in Tables 1, 2, with the omission of some of the finer details. In general, however, robot development progresses along cephalocaudal and proximo-distal learning directions. Whilst this is to be expected due to the choice of constraints, the experiment also demonstrates the efficiency in this learning pattern. Tables 6, 7 show the time taken for the robot to advance from the experiment's initial state to the final goal state is less than 4 h. This is possible because the constraints limit the size of the learning space, whilst the resultant ordering of stages generates a sequence whereby earlier behaviors create data for bootstrapping learning of later behaviors. For example, eye saccades provide data for learning of gaze control, which is in turn used as a basis for hand-eye coordination. Similarly, in the arm system, the staged increase of gaze field resolution enables mappings to be created that are initially very sparse, but which are then refined as resolution improves. Without bootstrapping, the high dimensionality of the space means considerably more learning will be required to reach a similar level of ability across all areas. For further material on this, for head and eye learning see Shaw et al. (2012), and for reaching see Law et al. (2014).

**Table 6:** **Behaviors observed on the iCub**.

**Behavior**	**Description**	**Time of appearance (min)**
Saccading	Eye movements only, trying to fixate on stimuli	0
Gazing	Eyes and head move to fixate on stimuli	20
Guided reaching	Successful and smoother reaches toward visual targets	60
Torso movement	Moves at waist to reach objects	120
Repeated touching	Repeatedly reaches out and touches objects	140
Pointing	Points to objects out of reach	160
Object play	Explores object affordances and actions	170
Stacks objects	Places one object on top of another	210
Learning ends	Experiment ends	230

**Table 7:** **Behaviors observed in simulation**.

**Behavior**	**Description**	**Time of appearance (min)**
Fetal babbling	General arm movements	0
Pre-reaching position	Moves hand to the side of the head before reaching	10
Swiping	Arms make swiping actions in the general direction of visual stimuli	10
Visually elicited reaching	Reaches toward visual targets with some success	20
Guided reaching	Successful and smoother reaches toward visual targets	30
Learning ends	Refined hand-eye coordination	90

Table 8 highlights the dimensionality issue, and shows how stages break down the mappings into manageable chunks. 15 degrees of freedom in the motor space are mapped to 15 dimensions in the sensory space, using seven core stages. Movements of the eyes, head, and torso are all mapped to the gaze space to provide visual orientation, but the series nature of the joints requires learning to follow the pattern eyes-head-torso. Reaching is learnt through four stages, with both arms learning in parallel. The four stages correspond to a shift from tactile and proprioceptive to visual mapping, followed by improvements in visual resolution.

**Table 8:** **Learning times using developmental processes**.

**System**	**Motor DoF**	**Sensory map dimensionality**	**Stages**	**Learning time (min)**
Eyes	3	3	1	20
Head	2	2	1	40
Tactile		1		
Torso	2	3	1	20
Arms	4*2	3*2	4	90

### 3.2. Impact of constraints

The second result concerns the impact of different constraints, and the timing of their removal, on learning. We use the eye and head components of the gaze system to illustrate the effects of both Type A and Type B constraints on the development of gaze control.

Gaze control is learnt in two stages: mapping of visual changes to the eye motors, and mapping of visual changes to the neck motors. In reflection of the human gaze system, a stabilizing ocular reflex causes the eyes to rotate to compensate for movements of the head, and maintain fixation on a stimulus. This prevents a direct mapping from neck motors to vision, as the eye reflex minimizes visual change. Therefore, the mapping must take into account changes in eye position and their known effect on visual stimuli, which requires a well developed eye mapping [for a detailed description of the gaze-learning algorithm see Law et al. (2013)].

We simulated the documented effect of poor muscle tone in the neck by imposing a constraint on head movement. We varied the time at which this constraint was released to model a Type A constraint, and varied the level of stimulation in the environment to model the effect of a Type B constraint. In the case of the Type A constraint, we compared the effect of reducing the head constraint at 10 min intervals over seven 1 h learning periods. In most cases this resulted in the eye and head systems learning *in parallel* for part of the learning period. In the case where the constraint was removed at time *t* = 0, an emergent constraint appeared with the head system failing to learn correct movements until the eye mapping was partially developed. Over the whole course of the learning period, this resulted in slower learning as both systems attempted to develop in parallel. In comparison, when the constraint lifting was delayed, the eye mapping was initially able to develop more rapidly on its own. When the constraint was eventually lifted, data from the eye map was available to support head learning, resulting in immediate learning of correc movements.

**Figure 6:**
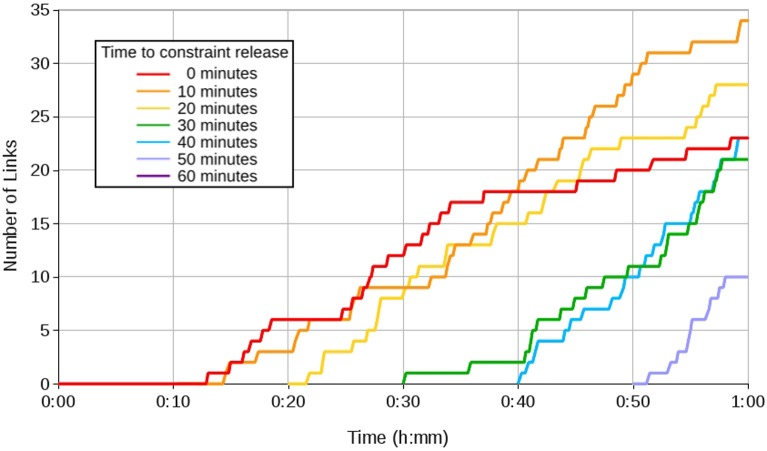
**Graph showing head learning with a Type A constraint lifted at 10 min intervals**.

Figure 6 shows the number of links in the head mapping learnt over time. The most links were learnt when the constraint was released between 10 and 20 min after eye learning had begun. This represents a trade-off between the level of eye control required to support head learning, and the remaining time available for learning.

In order to evaluate the impact of a Type B constraint on the eye and head development, we varied the level of stimulation within the environment. Previously a selection of static visual stimuli had been available for the robot to select as targets for saccade learning, however, in this case only a single static target was presented centrally in front of the robot. The effect of this was to limit the size of eye motor movements that could be made without losing sight of the target, thus limiting eye learning. Here, removal of the constraint on head movement enables the target to appear off-center of the eye, simulating its appearance at new locations.

**Figure 7:**
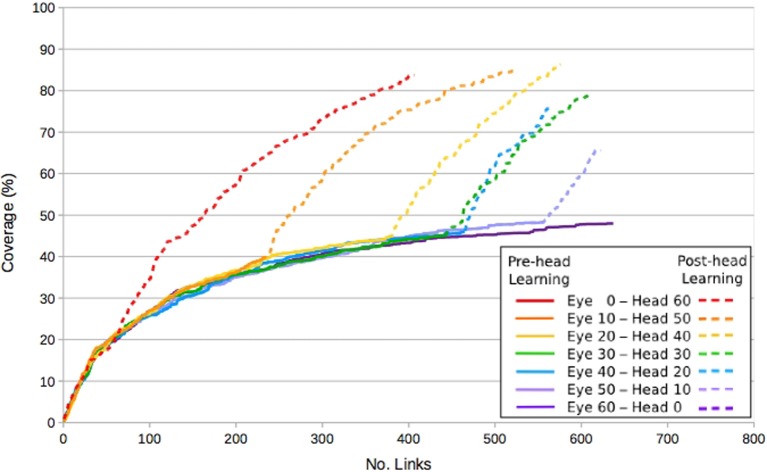
**Graph showing the effect of a Type B constraint on eye learning, when the head constraint is lifted at 10 min intervals**.

Figure 7 shows the coverage in the eye map, in terms of fields, as links are learnt. This is a measure of how much of the visual space can be reached by a known saccade. With only a single, stationary visual stimulus available, eye learning saturates at around 50% coverage. The effect of repositioning the stimulus can be clearly seen in the periods following the constraint removal, where coverage increases to around 80% without saturating. Further explanation of this phenomena can be found in Shaw et al. (2012).

These results show that both types of constraint impact on learning in significant ways. Maturational constraints prevent specific abilities, and limit the size of the learning space, whereas environmental constraints limit the complexity of the stimuli, and result in emergence of behaviors. Our experience is that both are required to drive efficient learning, but a balance is required. Too little constraint results in over stimulation, and problems in identifying correspondence, whereas over-constraint restricts and slows learning. Other mechanisms for releasing constraints are possible, e.g., Nagai et al. (2006) who compare an error measure method against fixed time scheduling.

### 3.3. Impact of bootstrapping between stages

The third result concerns the impact of bootstrapping between stages, that is, the value of priming new behavior with previously learnt data. We use the problem of learning arm reaching to illustrate this.

In order for the robot to be able to reach to and pick up objects, it is very desirable that the trajectory of the hand follows a reasonably direct route to the target, avoiding dangerous configurations, obstacles, and possible damage to the robot. To achieve this we developed a vector based reaching algorithm using an adaptation of our mapping technique. For each arm a 4-to-3 dimensional mapping is created between the joint space of the shoulders and elbow, and the gaze space. This enables learning the correspondence between arm postures and the corresponding position of the hand in the visual space. An important addition is the ability of fields in both maps to store vectors. These allow movement directions in the gaze space to be mapped to motor movements in the joint space by performing and observing small movements of the arm. As vectors are stored as part of the field data, movements are learnt in correspondence to particular arm poses. When reaching, combinations of vectors from the current or nearby postures can be used to move the hand in a desired direction.

Although it is possible to learn this mapping in one stage, using our novelty-driven motor babbling, we have found that learning can be made much more efficient by using multiple stages, and using data learnt in earlier stages to bootstrap learning in later ones.

We note that the eyes of the fetus do not open until 26 weeks after conception, and that any vision is likely to be very limited. However, arm movements and tactile perception appear at 7–9 weeks, and there is the possibility for early learning through proprioception and tactile feedback. To simulate this we created a very basic model of activity in the womb, through which simple arm movements are learnt using coarse proprioception. These are generated by motor babbling, and learning is triggered by tactile stimulation resulting from interaction with a modeled uterine wall. After 10 min of learning, a range of proprioceptive arm positions have been generated corresponding to these interactions, without any information on their position in space being stored. This data is then used to bootstrap hand-eye coordination and reaching.

**Figure 8:**
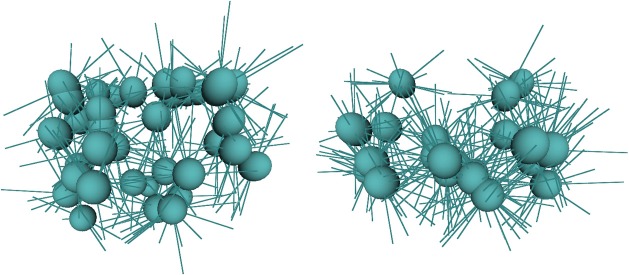
**Arm fields after 10 min of hand regard behavior**. The left image is with bootstrapping and the right is without bootstrapping.

In our experiment, we consider how even very primitive bootstrapping is important. During the immediate post-swiping stages in Table 5 the robot performs hand regard. That is, it looks to the position of the hand and makes small movements in several directions to generate the vector mapping described above. As the vectors are only valid for the pose in which they are learnt, hand regard must be performed over a range of poses for them to be useful to control reaching. The bootstrapping data from the previous stages provides a set of known positions at which hand regard can be performed and, due to the ballistic character of much of the motor babbling behaviors, the locations tend to be at the extremities of the operating (reachable) space. This distribution in space is an advantage because movements between the locations provide a good covering of the space, whereas without this data, hand regard would tend to cluster around the central area and take much longer to explore the extremities. Figure 8 shows images of the arm fields generated after 10 min of hand regard and reach learning. Using bootstrapping produces 36 fields with an average of 8.4 vectors per field, while without bootstrapping there are 22 fields with an average of 15.9 vectors per field. This shows how learning without bootstrapping is centered around a smaller set of configurations. Further data on the stages of reach learning can be found in Law et al. (2014).

Another aspect of staged transfer is seen in the removal of the gaze field constraint shown in Table 4. When vision is first used the generated fields are restricted to a large radius (0.7) and hence the covering of space is coarse. After 15 fields are produced the field size constraint is lifted (to radii = 0.2) and then the number of fields increase to 33 before the next stage. Thus the spatial covering becomes more exact and more accurate movements can be made. This differentiation of coarse or diffuse values into finer resolutions is seen in other developmental studies, e.g., regarding visual immaturity, Nagai et al. (2011) have shown how early sensorimotor associations formed during periods of poor discrimination can continue to be important when much finer discrimination has been achieved.

**Figure 9:**
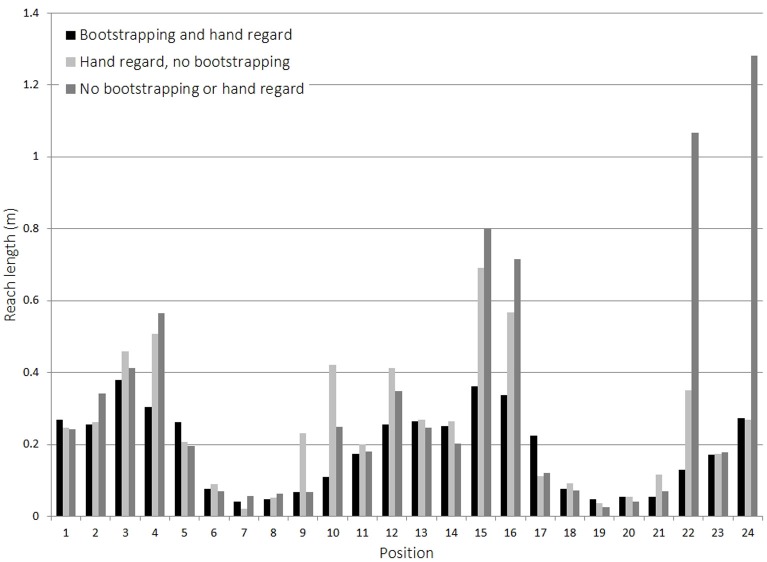
**Effect of bootstrapping on reach learning**.

The results in Figure 9 show the distances covered by the hand when reaching to a set of predefined targets using learnt vector-based reach mappings. The three different data sets correspond to three different learning strategies: the first uses the method described above, performing hand regard at the poses in the bootstrapping data. The second ignores the bootstrapping data, but performs hand regard at positions it encounters whilst trying to reach to a target. The third uses neither bootstrapping nor hand regard, and only learns vectors corresponding to movements it makes whilst trying to reach to the targets. The main difference between these last two is that with hand regard the robot learns vectors in multiple directions, whereas without hand regard it can only learn vectors corresponding to the direction of motion. If no suitable vector to direct reaching was known, then a random movement is made. Learning was conducted for the same duration for each approach and then the mappings used to control reaches to 24 target positions, 12 for each arm, distributed throughout the robot's reachable space.

The results display the clear advantage in using bootstrapping data from previous stages. Table 9 illustrates this by using deviation from the most direct path as an error measure. By comparing the average distance covered by the hand to the ideal straight-line path, of the three approaches, the one using bootstrapping resulted in a near ideal case.

**Table 9:** **Reach length comparison**.

**Learning method**	**Average hand trajectory length compared to the direct path (%)**
Bootstrapping and hand regard	107.5
Hand regard, no bootstrapping	149.5
No bootstrapping or hand regard	179.5

### 3.4. Long-term memory for identifying novel events

Our fourth significant result shows how a memory of experienced actions enables appropriate responses to novel events and thus provides a framework for the emergence of new action skills.

As described in section 2.4, without a long-term memory the sensorimotor mappings can only support repeated actions over short-term events, hence we use a schema learning mechanism that can record more complex actions and their consequences (Sheldon and Lee, 2011). A schema is a structure that encodes the context in which an action may be performed together with the action detail and its result, or more formally: <preconditions : action : postconditions>. An example schema for touching an object, *A*, at a specific location can be written:

<A at (35, 66) : Reach to (35, 66) : A at (35, 66), Hand at (35, 66), Touching A>

Schemas are created when an action is first performed, using the action and a set of observations. They also carry excitation values and data relating to the probability of their occurrence. Schema recall occurs when a new sensory event is detected and the schemas are scanned to find those that most match the current situation. This is achieved by exciting the schemas using a combination of the novelty of the current experience and their similarity to past experiences. The level of excitation increases with the novelty of the current sensation and the similarity to a remembered sensation (see Sheldon, 2012, for full details of the schema creation, matching, and generalization algorithms).

**Figure 10:**
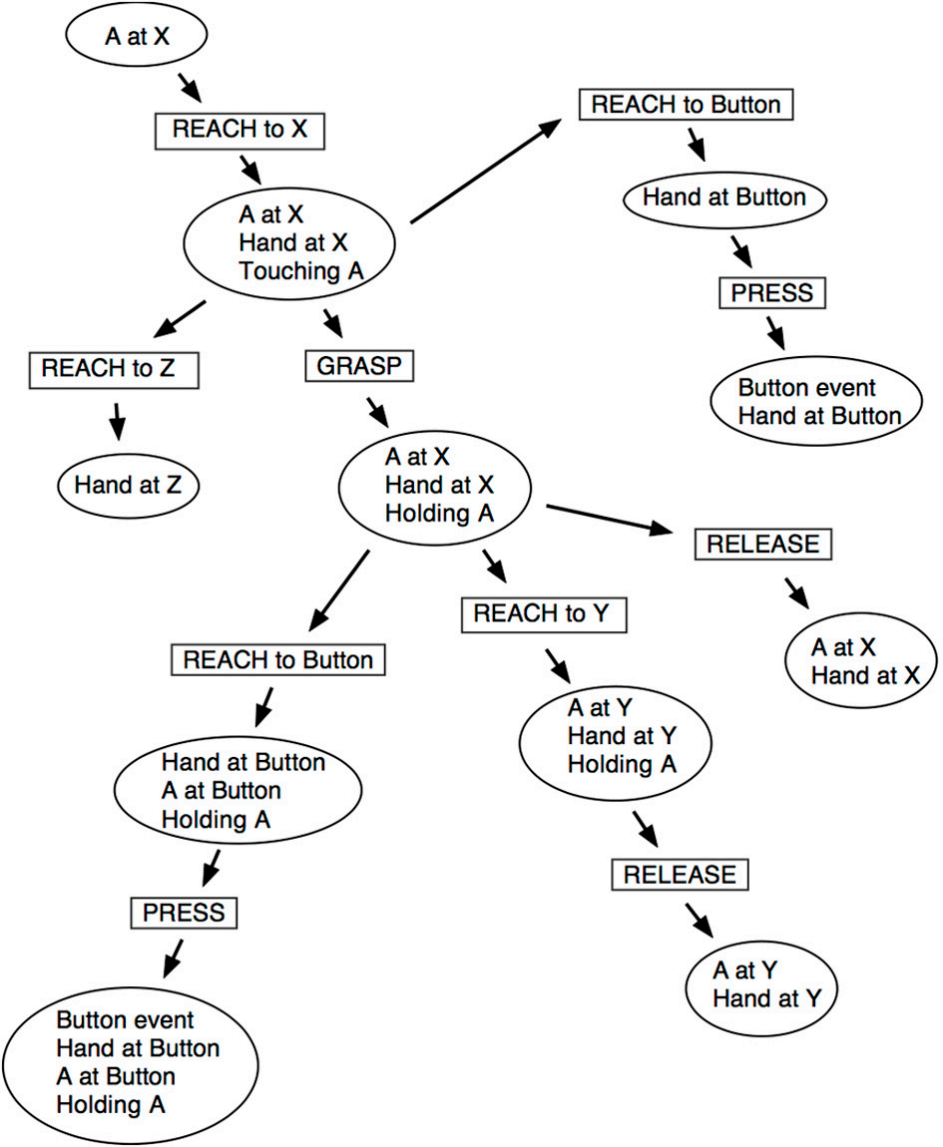
**A schematic map of some schema chaining possibilities**. Rectangular boxes represent actions or state transitions and elliptical boxes represent different states known to the robot.

Just like stimuli, schema excitation values decrease as they are used. This means newly discovered schemas are more likely to be repeated and tested. Schema probability values track the likelihood of the schema succeeding and the more excited and predictable schemas are selected in preference to less excited and unpredictable ones. The result is that, initially, simple but reliable schemas are selected and explored. However, as their excitation levels drop more complex and potentially useful behaviors come to the fore. This promotes exploration when there are few immediate novelties, and can result in unexpected behavior. For example, in the later stages of the experiment the iCub had learnt schemas for reaching, pressing buttons and grasping objects. The iCub next learned that it could reach to, and grasp, an object, and that it could move that object by reaching to new locations. Figure 10 illustrates how these actions can occur. The diagram indicates some possible states of the sensory data that schema actions can cause to change. At the top-left in Figure 10 an object, *A*, is known to be located at position *X*. The sequence of schema applications along the diagonal toward bottom-right correspond to grasping an object and moving it to another place. Several other schemas are illustrated: the earlier schema of grasping but not moving an object is at center-right; and the pressing action is shown at top-right.

After the reaching-whilst-holding schema had become established, the iCub discovered that it could conduct a pressing action whilst holding an object; this was composed from the two above unrelated actions and is seen at left-bottom in Figure 10,^7^. The motivational conditions that caused this, through the excitation and matching of prior experience to new situations, demonstrated how two unrelated actions may be combined to form new skills, and opens up the exciting prospect of learning tool use.

An important property of the schema framework is the ability to make generalizations. The generalization mechanism produces schemas containing parameters which can be populated based upon the current experiences of the robot when being executed. Beyond simply determining which aspects of the schema may be interchangeable with other values as many existing schema systems do, this mechanism attempts to find generalizable relationships between the preconditions, the action and the post-conditions of a schema. This allows the generalized schemas which are produced as a result of this process, to represent the agent's hypotheses about how an interaction may work at a more abstract level (see Sheldon, 2012, for further details).

Along with stages, generalization offers the means to reduce complexity in the learning environment. Table 10 shows the number of schemas learnt to enable the robot to be able to reach and touch objects at any location within its workspace, or point to those out of reach. Without stages all combinations of stimuli and events create potential schemas, and so the prohibitive numbers of robot actions mean simulation is necessary to investigate this aspect. These results show that staged learning dramatically reduces the number of schemas that are learnt by a simulated robot, and that combining a staged approach with the generalization mechanism reduces this number even further. Interestingly, experiments on the real robot produce even fewer schemas due to an additional constraint; the visual range of the cameras used on the real robot was more restricted than that in the simulator model.

**Table 10:** **Effect of development and generalization on schema production**.

**Scenario**	**Number of schemas produced**
Generalization only (Simulated Robot)	19,244
Stages only (Simulated Robot)	347
Stages, generalization (Simulated Robot)	227
Stages, generalization (Physical Robot)	115

Table 11 gives an example of a sequence of schemas learnt on the iCub, and the times of their creation^8^. Initially the robot has access to the primitive sensorimotor actions contained in the learnt mappings, which include gazing and reaching. It also has preprogrammed reflex grasp and button-pressing actions.

**Table 11:** **Schema discovery on the iCub**.

**Time (mm:ss)**	**Preconditions**	**Action**	**Postconditions**	**Description**
00:18	Green object at (17.5, 72.4)	Reach to (17.5, 72.4)	Hand at (17.5, 72.4)	New touch schema
			Green object at (17.5, 72.4)	
			Touch sensation	
00:50	$z color object at ($x,$y)	Reach to ($x,$y)	Hand at ($x,$y)	Generalized touch schema
			$z color object at ($x,$y)	
			Touch sensation	
01:56	Green object at (17.5, 72.4)	Grasp	Hand at (17.5, 72.4)	New grasping schema
	Touch sensation		Green object at (17.5, 72.4)	
			Holding object	
02:01	$z color object at ($x,$y)	Grasp	Hand at ($x,$y)	Generalized grasp schema
	Touch sensation		$z color object at ($x,$y)	
			Holding object	
02:19	Hand at (17.5, 72.4)	Reach to (8.8, 62.6)	Hand at (8.8, 62.6)	New transport schema
	Green object at (17.5, 72.4)		Green object at (8.8, 62.6)	
	Holding object		Holding object	
02:36	Hand at ($x,$y)	Reach to ($u,$v)	Hand at ($u,$v)	Generalized transport schema
	Green object at ($x,$y)		Green object at ($u,$v)	
	Holding object		Holding object	
03:42	Hand at (17.5, 72.4)	Release	Hand at (17.5, 72.4)	New release schema
	Green object at (17.5, 72.4)		Green object at (17.5, 72.4)	
	Holding object		Touch sensation	

The $ notation specifies variable bindings, in left to right order.

At the outset the robot is presented with a green object. As the most novel stimuli this triggers available actions: first gaze, then reach. At 0:18, the robot receives tactile feedback from this action, which results in the generation of a new schema for touching a green object at that location. The excitation of this schema causes the action to be repeated. Noise in the system creates some subtle variation, and leads to the generation of some similar schemas. At 0:50 these are generalized into a schema for touching any colored object at any location (note that in this experiment we have set the requirements for generalization to a minimum to enable fast learning). The robot then tests the generalization by repeating the action. At 1:45 the excitation of the touching schemas have dwindled, and the grasp action now has the highest excitation. This is due to the similarity between the existing touch sensation and the recorded touch sensation triggered by closing the hand on itself. At 1:56 the robot generates a new schema for grasping a green object at that location, and this is quickly followed by the generalized version due to the similarity with the existing generalized touching schema. The robot cannot re-grasp, and so reaching again becomes the most excited action. At 2:19 the robot has moved its hand to a new position whilst still holding the object. This creates a new schema for moving an object that, following more repetition, becomes generalized at 2:36. At 3:32, after further repetition, the most excited option becomes the press action. This is particularly interesting as the robot is still holding the object, and provides the opportunity for learning basic tool use. However, in this instance the action does not cause a change in the world state, so no schema is generated. Finally, the release action becomes most exciting, and so the robot drops the object, learning the “release” schema.

## 4. Discussion

This paper has described a longitudinal experiment in robotic developmental learning. Starting from a state of uncontrolled motor babbling, the iCub robot displayed a developmental progression, passing through several distinct behavioral stages, until skilled visio-motor behavior, involving reaching and manipulation of objects, was achieved. The result tables show the various learning times required for the robot to reach repeatable performance with reasonable accuracy and the total time for the whole process is less than 4 h. Such fast learning rates are crucial in real robot systems where online learning is essential, and training through many thousands of action cycles is quite impossible. This performance, which is typical of all our experiments, show that developmental learning algorithms offer serious potential for future real-time autonomous robots that must cope with novel events.

Whilst most comparable work in developmental robotics is focused on mechanisms within a single developmental stage, we are investigating longitudinal development and the transitions between multiple stages of behavior. Our methodology has been to implement the various subsystems in a way that facilitates their interaction, guided by the psychological literature to provide insight and inspiration. By closely following the stages evident in infancy, we find that learning in the robot is well directed along a trajectory that simplifies and reduces the amount of learning required. Furthermore, just as with infants, these trajectories will be similar but never exactly the same. In the early stages, where sensor and motor activity is being coordinated, learning is affected by variation in stimuli and motor babbling. In the later stages, the trajectory of schema development is dependent on the learnt primitive skills, the initial excitation of schemas, and the environment. Therefore trajectories can, and do, vary in their appearance across experiments.

Imposing carefully selected constraints can very effectively reduce the complexity of learning at each stage, with earlier stages providing valuable data for bootstrapping later stages. The experiments show some of the conditions for the interaction of different constraints (maturational or environmental) that can enhance learning rates. Whilst we have imposed the general order of constraints to structure the earlier stages of development, their release has been determined by internal measures, allowing the variations described above. Furthermore, we have shown how the early and late release of constraints impacts on development. We have also shown how stages may emerge based on environmental factors, or through experience, as shown by our schema experiments. Although it is possible to trigger constraint release by various means, as is sometimes necessary in experiment, we believe that emergent states based on current levels of development may account for this process without recourse to specific mechanisms. This requires further investigation.

In following the longitudinal approach it becomes necessary to recognize that any current stage under study is conditioned by the previous stages which may feed in structures and experience that can influence the resultant performance. This means the earliest stage possible should be the start point and although we originally started with the newborn we realized that the fetal stage can make an important contribution in the bootstrapping sense. It seems the early sensorimotor organization occurring at this stage could be of considerable significance for the development of later abilities.

We have drawn on various sources for guidance on these issues, these include: the emergence of stereotypical movements and actions in the prenatal period (Mori and Kuniyoshi, 2010; Yamada and Kuniyoshi, 2012); learning to control saccades and gaze shifts (Srinivasa and Grossberg, 2008); and the emergence of stereotypical reaching behavior (Schlesinger et al., 2000), including the benefits of the ordered release of constraints (Savastano and Nolfi, 2012). Other relevant work includes that of Grupen and colleagues who were amongst the first to recognize the potential of the cephalocaudal progression of infant development as a robotic technique (Coelho et al., 2001; Grupen, 2005; Hart and Grupen, 2013), and the proximo-distal heuristic has been widely recognized, e.g., Elman (1993). Other key projects are investigating periods of cognitive growth through a variety of robotic platforms and models (Asada et al., 2009; Mori and Kuniyoshi, 2010), and reaching has received particular attention regarding the staged release of constraints (Savastano and Nolfi, 2012), their impact (Ramırez-Contla et al., 2012), and possible emergence (Stulp and Oudeyer, 2012).

Another distinct feature of the results is the use of motor babbling behavior to drive learning. Whilst most other similar systems use goal-driven and error-reducing methods, we note that goals and errors are usually specified by the system designers. We consider it important to investigate general action and open-ended exploratory/goal-finding behavior. In this context goals and errors are to be discovered or given significance by the agent itself. The simple novelty algorithm combined with motor babbling provides an effective exploratory learning mechanism that generates much pertinent data for learning about sensorimotor experience. Motor babbling is a form of spontaneous action and the excitation method applies to both single actions and action sequences during schema selection. This means that novel action patterns can emerge, as seen in the experiment, and this type of behavior is very reminiscent of infant play, which is also an exploratory goal-free behavior. Play has been long recognized as a critical and integral part of child development and the importance of novelty-driven play in infant development is well established (Bruner et al., 1976). We view play as an extension of motor babbling behavior, and schemas as the substrate to support this process. This hypothesis is described further in Lee (2011).

To summarise, this experiment has provided a demonstration of longitudinal development as a particularly fast and effective sensory-motor learning technique. Constraints have been used to shape infant-like behavior development, and we find these have an important role in speeding learning in robotic models. In particular, data learnt at a more constrained stage can bootstrap later learning, leading to improved performance. Finally, the combination of very simple novelty detection mechanisms and intrinsic babbling algorithms are, at least, sufficient to drive learning of early sensory-motor coordination and basic skill acquisition.

### Conflict of interest statement

The authors declare that the research was conducted in the absence of any commercial or financial relationships that could be construed as a potential conflict of interest.

## Footnotes

^1^The iCub is designed to be closely modeled on infant anatomy. For example, the eyes can saccade at speeds approaching human performance; we set the saccade velocity to be 80 degrees/s.

^2^In our system visual detection is based on objects represented as patches of color. An object, consisting of a cluster of pixels of minimum radius 5 pixels, is considered to have changed if 20% of the pixels change. Prediction or expectation is determined by the sensory data matching, or partially matching, an existing stored schema.

^3^Each field can hold a range of variables, effectively providing a 2.5D representation. This is how space is represented with depth as a field value.

^4^The version angle is the combined pan angle of the two cameras.

^5^A time lapse video of the longitudinal process on the iCub can be found at http://youtu.be/OhWeKIyNcj8

^6^Videos of the robot performing some of these stages, with basic reaching and torso movements, can be found at http://youtu.be/_ZIkbU8FZbU and http://youtu.be/3zb88qYmxMw. Full reaching and torso control is shown in http://youtu.be/OhWeKIyNcj8

^7^A video of this behavior can be found at http://youtu.be/VmFOoobKd9A

^8^A video of this sequence can be seen at http://youtu.be/3zb88qYmxMw
